# New Treatment Regimens, New Drugs, and New Treatment Goals for Lupus Nephritis

**DOI:** 10.3390/jcm14020584

**Published:** 2025-01-17

**Authors:** Giovanni M. Rossi, Augusto Vaglio

**Affiliations:** 1 Nephrology Unit, University Hospital of Parma, 43126 Parma, Italy; 2 Nephrology and Dialysis Unit, Meyer Children’s Hospital IRCCS, 50139 Florence, Italy; 3 Department of Biomedical, Experimental and Clinical Sciences “Mario Serio”, University of Florence, 50121 Florence, Italy

**Keywords:** lupus nephritis, belimumab, voclosporin, obinutuzumab, cd20, proteinuria

## Abstract

Lupus nephritis is one of the most severe manifestations of systemic lupus erythematosus, affecting roughly 40% of all lupus patients. With the introduction of cyclophosphamide and mycophenolate mofetil, outcomes have dramatically improved. However, 10% of patients still progress towards end-stage kidney disease, which carries an elevated mortality rate. In recent years, several novel agents have been approved for use or have shown preliminary evidence of efficacy in lupus nephritis. These agents include belimumab, voclosporin, and obinutuzumab, among others. Efficacy has also been demonstrated in recent trials combining older drugs. However, determining which patients would benefit the most from novel agents or combined drug regimens and whether these drugs might serve as an alternative to current remission-induction drug regimens rather than as add-on therapies remain unresolved issues. In this review, we will explore the current evidence regarding the efficacy of novel agents.

## 1. Introduction

Lupus nephritis (LN) is a common manifestation of systemic lupus erythematosus (SLE) and, along with central nervous system involvement, is the most severe in terms of the risk of end-organ damage (i.e., end-stage kidney disease, ESKD) and mortality. The advent of cyclophosphamide in the 1970s and mycophenolate mofetil in the 2000s, which became the standard of therapy along with glucocorticoids, dramatically improved renal outcomes, leading to the currently most consistently reported fraction of roughly 5–10% of all LN patients now reaching ESKD [1]. Research in this field shifted over the years from the need for more efficacious and steroid-sparing regimens (cyclophosphamide) to lowering the burden of treatment-related side effects while retaining efficacy (low-dose cyclophosphamide regimens, mycophenolate mofetil, combined multidrug regimens) [2,3,4,5,6,7]. In recent years, novel agents were tested as add-ons to the standard of care in robust phase III randomized controlled trials, with the introduction of belimumab and voclosporin to our current arsenal [8,9,10]. Anti-CD20 monoclonal antibodies were also investigated, with the introduction of rituximab as a choice for refractory patients despite the failure of phase III trials [11,12]. This approach is likely to change further based on the efficacy of obinutuzumab, a novel anti-CD20, as an add-on to the standard of care (SOC), shown in phase II trials and currently under investigation in phase III trials [13,14]. With the advent of new drugs, the focus of research has broadened to concurrently include the need for (i) the minimization of corticosteroid exposure, (ii) the minimization of treatment-related adverse effects, particularly infections, and (iii) patient-tailored treatment. In light of the need to address the significant proportion of patients still reaching ESKD, traditional views of remission-induction and maintenance-induction treatment regimens and trial-prespecified renal outcomes (e.g., proteinuria) have been questioned, highlighting the need for research and treatment strategies aimed at preserving long-term renal function.

LN is characterized by immune complex deposition and complement activation in the renal glomeruli, which drives inflammation, proteinuria, and progressive renal dysfunction. In contrast, SLE patients without LN often experience milder disease phenotypes, dominated by systemic features such as fatigue, arthralgia, and mood disturbances, aligning with the Type 2 SLE symptoms defined in the Type 1 and 2 SLE Model. These patients typically report persistent symptoms that significantly impair quality of life despite the absence of overt organ damage. Clinically, patients with LN frequently present with more severe disease activity, as reflected in higher SLE Disease Activity Index (SLEDAI) scores and systemic involvement, including active serology such as hypocomplementemia and elevated anti-dsDNA antibodies. In contrast, SLE without LN is more commonly associated with milder relapsing-remitting disease courses, with approximately 70% of such cases achieving periods of remission compared to lower rates in LN cohorts. Furthermore, the evolution of LN is often marked by episodes of acute nephritic flares. By comparison, SLE patients without LN rarely develop life-threatening complications, with their disease progression largely dominated by cumulative fatigue and chronic pain syndromes [15,16].

The disparity in clinical outcomes also extends to therapeutic approaches. While SLE without LN is typically managed with antimalarials, glucocorticoid-sparing strategies, and symptomatic treatments for Type 2 symptoms, LN requires more intensive immunosuppression. Maintenance therapies for LN aim to reduce the risk of flare and preserve renal function, whereas non-LN SLE management prioritizes the control of systemic inflammation and the mitigation of long-term quality-of-life impairments.

Mortality rates in systemic lupus erythematosus (SLE) remain significantly elevated, with patients facing a threefold higher risk of death compared to the general population. Notably, survival rates have stagnated since the mid-1990s, and substantial disparities persist globally due to unequal access to healthcare. Approximately two-thirds of fatalities are linked to treatment-related complications, such as infections stemming from prolonged corticosteroid use or potent immunosuppressive therapies, while the remaining third is attributed to active SLE disease [17].

In this review, we will summarize current evidence regarding newly introduced drugs in LN, discuss their place in the management of LN patients with respect to current treatment guidelines, and discuss the controversies surrounding surrogate renal outcomes in trial design.

## 2. Current Eular and Kdigo Guidelines

The most recent LN guidelines from EULAR and KDIGO kept a traditional treatment approach based on the ISN/RPS histological classification of LN and largely concur with specific recommendations (Table 1).

According to the EULAR 2023 guidelines, patients with active proliferative LN should be treated with low-dose intravenous cyclophosphamide or mycophenolate combined with glucocorticoids, including initial pulses of intravenous methylprednisolone followed by oral tapering. Combination therapy with belimumab or calcineurin inhibitors, such as voclosporin or tacrolimus, may be considered alongside mycophenolate or cyclophosphamide. Maintenance therapy should continue for at least three years, with mycophenolate or azathioprine replacing cyclophosphamide for those initially treated with it. For high-risk patients, characterized by reduced glomerular filtration rate (eGFR < 45 mL/min/1.73 m^2^), cellular crescents, fibrinoid necrosis, or severe interstitial inflammation, high-dose intravenous cyclophosphamide (NIH regimen) with pulse methylprednisolone may be appropriate. In cases of sustained remission, treatment tapering should prioritize glucocorticoid withdrawal. For class I and II LN, no recommendations are given. For class V, the cut-off for immunosuppressive treatment is >1 g/24 h proteinuria despite maximal RAAS blockade with or without SGLT2i, which is questionable considering the same guidelines state that the treatment regimen of choice for pure class V LN is controversial due to the under-representation of class V patients in clinical trials, and should be tailored to the individual patient’s characteristics, i.e., based on their risk for kidney failure. All LN patients should receive 5 mg/kg hydroxychloroquine daily. Rituximab is not recommended by EULAR 2023 guidelines for LN but “should be considered” in all cases of organ-threatening disease, which obviously includes LN with a high risk of progression towards ESKD [18].

According to the 2024 KDIGO guidelines, for the initial treatment of active Class III or IV LN, with or without a membranous component, glucocorticoids are recommended alongside one of the following: mycophenolic acid analogs (MPAA), low-dose intravenous cyclophosphamide, a combination of belimumab with MPAA or cyclophosphamide, or MPAA with a calcineurin inhibitor (CNI) for patients with preserved kidney function (eGFR > 45 mL/min/1.73 m^2^). Intravenous cyclophosphamide is preferred for patients with adherence challenges, while MPAA is favored for those at a high risk of infertility due to prior cyclophosphamide exposure. CNIs may be prioritized for patients with nephrotic-range proteinuria or intolerance to standard regimens, and triple therapy with belimumab is an option for patients with repeated kidney flares or advanced chronic kidney disease. Alternative therapies, such as azathioprine or leflunomide, may be used when standard drugs are unavailable or unsuitable, though with potentially reduced efficacy. Maintenance therapy involves tapering glucocorticoids to the lowest effective dose, with MPAA as the preferred agent (MMF 750–1000 mg or MPA 540–720 mg twice daily) for a total treatment duration of at least 36 months. Azathioprine is an alternative for patients intolerant to MPAA or planning pregnancy. Triple immunosuppressive regimens may be continued into maintenance therapy, and CNIs, mizoribine, or leflunomide can be considered if MPAA or azathioprine are unsuitable. Glucocorticoid discontinuation may be considered after 12 months of complete renal response. Newer therapies, including rituximab, may be explored for refractory disease. The treatment of class I and II is guided by extrarenal manifestations and/or the presence of nephrotic-range proteinuria, which warrants ultrastructure examination to exclude lupus podocytopathy, which should be treated with glucocorticoids. The treatment of class V differs from the 2023 EULAR recommendations in that a combined glucocorticoids plus MPAA or CyC regimen are recommended when nephrotic range proteinuria despite RAAS is presentor can be considered when proteinuria worsens and/or its complications (edema, thrombosis, dyslipidemia) arise [19].

## 3. New Combinations of Old Drugs: The “Multitarget” Approach

The so-called multitarget treatment involves a combination of tacrolimus, MMF, and prednisone. The two randomized trials evaluating this regimen were conducted in an Asian population, both for induction and maintenance of remission [6,7].

The induction trial compared a combination regimen of tacrolimus (4 mg/day), mycophenolate mofetil (MMF, 1 g/day), and glucocorticoids to intravenous cyclophosphamide (IVCY), enrolling 368 patients with biopsy-proven active LN (class III, IV, V, III + V, or IV + V). Inclusion criteria required proteinuria ≥ 1.5 g/day and serum creatinine ≤ 3.0 mg/dL. Both groups received high-dose methylprednisolone pulses (0.5 g/day for 3 days) followed by a tapered prednisone course starting at 0.6 mg/kg/day.

At week 24, complete remission (CR) rates were significantly higher in the group treated with the combination regimen compared to the IVCY group (45.9% vs. 25.6%; difference, 20.3%; *p* < 0.001). Overall response rates (CR and partial remission) were also superior in the combination regimen group (83.5% vs. 63%; difference, 20.4%; *p* < 0.001), with a faster time to respond (median 8.9 weeks vs. 13.0 weeks; *p* < 0.001). Adverse event rates were comparable (50.3% vs. 52.5%). These findings demonstrated the superiority of the combination approach in achieving remission with a similar safety profile in this specific population.

Following the induction phase, an 18-week maintenance study included 206 patients who achieved CR or partial remission. Patients continuing the combination treatment received tacrolimus (2–3 mg/day), MMF (0.5–0.75 g/day), and low-dose prednisone (10 mg/day), while the control group received azathioprine (2 mg/kg/day) with prednisone. The primary outcome was renal relapses. Renal relapse rates were low and similar between the combination group and the azathioprine group (5.47% vs. 7.62%; HR, 0.82; *p* = 0.74). However, the group continuing combination treatment experienced fewer adverse events (16.4% vs. 44.4%; *p* < 0.01) and lower withdrawal rates due to adverse events (1.7% vs. 8.9%; *p* = 0.02). eGFR and serum creatinine levels remained stable in both groups. These results indicate that the continued use of combination treatment during maintenance effectively prevents relapses while reducing adverse events.

Since these trials were conducted on an exclusively Chinese population, the generalizability is limited, but one might speculate that, if results were confirmed in other populations, compliance to a triple-drug regimen might be an issue; therefore, a careful evaluation of patients who are most likely to be adherent to treatment would be required.

## 4. New Drugs: Belimumab, Voclosporin, Obinutuzumab, and Anifrolumab

### 4.1. Belimumab

Belimumab is a fully human recombinant monoclonal antibody of the IgG1 lambda (IgG1λ) isotype that targets and inhibits the soluble B-lymphocyte stimulator (BLyS), also known as B-cell activating factor (BAFF). BLyS is a protein crucial for the survival and differentiation of B lymphocytes in immunoglobulin-producing plasma cells. Elevated BLyS levels in systemic lupus erythematosus (SLE) promote the survival of autoreactive B cells, contributing to disease progression through increased auto-antibody production. By antagonizing BLyS, belimumab reduces the survival of autoreactive B cells, mitigates auto-antibody production, and diminishes disease activity. Its fully human design minimizes the risk of immunogenicity, making it suitable for long-term use [20]. The BLISS-LN phase 3 trial [8] enrolled 448 adults with a biopsy-proven diagnosis of proliferative LN with or without a membranous component or Class V LN within 6 months before randomization. Patients received either intravenous belimumab (224 patients) or placebo (224 patients) in addition to SOC. The latter consisted of either MMF (target dose 3 g/day) or a cyclophosphamide-azathioprine regimen. Both regimens included glucocorticoids tapered to ≤10 mg/day by week 24. Belimumab 10 mg/kg or placebo was administered on days 1, 15, 29, and every 28 days until week 100. The trial population had a mean age of 33 years, with a large predominance of female subjects (88%). Ethnicity composition included 50% Asian, 33% White, 14% Black, and smaller percentages of other ethnic groups.

The primary outcome, a primary efficacy renal response at week 104 (defined as a urinary protein-to-creatinine ratio ≤ 0.7, an estimated glomerular filtration rate [eGFR] no worse than 20% below pre-flare levels or ≥60 mL/min/1.73 m^2^, and no rescue therapy use), was achieved by 43% in the belimumab group compared to 32% in the placebo group (odds ratio [OR], 1.6; 95% confidence interval [CI], 1.0–2.3; *p* = 0.03). Secondary outcomes included a complete renal response at week 104 (protein-to-creatinine ratio < 0.5 and eGFR no worse than 10% below pre-flare or ≥90 mL/min/1.73 m^2^), observed in 30% of belimumab recipients versus 20% in the placebo group (OR, 1.7; 95% CI, 1.1–2.7; *p* = 0.02).

A post hoc analysis of the BLISS-LN [21] demonstrated that patients treated with belimumab experienced a significantly slower annual decline in eGFR compared to those receiving placebo (−0.99 vs. −3.18 mL/min/1.73 m^2^/year; *p* = 0.0407). Additionally, belimumab significantly reduced the risk of a sustained 30% and 40% decline in eGFR, markers strongly associated with ESKD progression. Specifically, the sustained 30% decline occurred in 3.6% of belimumab-treated patients versus 11.2% of placebo-treated patients (OR, 0.29; *p* = 0.0042), while a sustained 40% decline occurred in 1.8% versus 6.7% (OR, 0.25; *p* = 0.0166).

The post hoc analysis of the BLISS-LN trial revealed that belimumab significantly improved renal outcomes, with notable differences observed based on baseline proteinuria levels (<3 g/g vs. ≥3 g/g). Among patients with baseline proteinuria <3 g/g, belimumab increased the Primary Efficacy Renal Response (PERR) rate to 54.5% compared to 33.6% in the placebo group (OR, 2.44; 95% CI, 1.46–4.08). In contrast, in patients with baseline proteinuria ≥3 g/g, belimumab did not demonstrate a significant difference in PERR rates (26.4% vs. 30.4%; OR, 0.85; 95% CI, 0.44–1.63).

For the complete renal response (CRR), the benefits of belimumab were also more pronounced in patients with proteinuria <3 g/g, achieving a CRR rate of 38.9% versus 22.1% with placebo (OR, 2.18; 95% CI, 1.26–3.78). In contrast, in the ≥3 g/g group, CRR rates were 17.6% for belimumab and 16.3% for placebo (OR, 1.20; 95% CI, 0.54–2.64).

Additionally, belimumab reduced the risk of a sustained 30% decline in eGFR across both proteinuria thresholds but with a greater magnitude in the <3 g/g group. In patients with proteinuria < 3 g/g, 12.9% in the placebo group experienced a sustained 30% eGFR decline, compared to 6.5% in the belimumab group (HR, 0.42; 95% CI, 0.23–0.75). For those with proteinuria ≥ 3 g/g, the rates were 31.5% and 19.8%, respectively (HR, 0.62; 95% CI, 0.34–1.12).

These results underscore the differential efficacy of belimumab based on baseline proteinuria levels, with a more robust renal protection observed in patients with proteinuria <3 g/g, while also demonstrating potential benefits in slowing the progression to ESKD in patients with higher proteinuria.

The main additional benefit of belimumab to SOC in LN seems to be the decreased rate of flares, therefore making it an ideal choice for LN patients with a history of relapsing nephritis and/or progressive CKD.

### 4.2. Voclosporin

Voclosporin, an oral calcineurin inhibitor (CNI) derived from cyclosporine, features a cyclic undecapeptide structure with a single carbon extension at amino acid-1, enhancing its efficacy, metabolic stability, and safety while eliminating the need for therapeutic drug monitoring. It forms a complex with cyclophilin A, which inhibits calcineurin, a key enzyme in T-cell activation. This structural modification not only increases potency but also shifts the primary metabolism to amino acid-9, producing the less potent IM9 metabolite with a low metabolite load. In contrast to cyclosporine, which generates nephrotoxic metabolites like AM1 and AM19, voclosporin’s reduced metabolite production is expected to minimize competitive inhibition and the risk of nephropathy [22]. The AURORA 1 trial [9], a phase 3, randomized, placebo-controlled study, included 357 patients with active lupus nephritis, comparing voclosporin (179 patients) with placebo (178 patients), each alongside MMF and low-dose steroids. Eligible patients had a diagnosis of SLE with active LN according to the American College of Rheumatology criteria. Inclusion required a kidney biopsy within the past two years confirming lupus nephritis class III, IV, or V (either alone or in combination with class III or IV), and evidence of active disease defined as UPCR ≥ 1.5 mg/mg for class III/IV or ≥2 mg/mg for pure class V. Patients with an eGFR ≤ 45 mL/min/1.73 m^2^ at screening were excluded. The standard treatment consisted of MMF at a dose of 2 g/day (with allowance for increases up to 3 g/day if necessary) and rapidly tapered low-dose glucocorticoids starting at 20–25 mg/day prednisone (or equivalent) and reduced to ≤2.5 mg/day by week 16.

At 52 weeks, 41% of patients in the voclosporin group achieved a complete renal response (CRR), defined as a UPCR ≤ 0.5 mg/mg, eGFR ≥ 60 mL/min/1.73 m^2^ or no reduction >20%, and no rescue medication use, compared to 23% in the placebo group (odds ratio [OR], 2.65; 95% CI, 1.64–4.27; *p* < 0.0001). Proteinuria reductions were rapid, with 97% of voclosporin patients achieving at least a 50% reduction in UPCR versus 76% of placebo patients.

The AURORA 2 trial [10] extended the treatment to three years, enrolling 216 patients from AURORA 1. Voclosporin sustained efficacy with a CRR rate of 50.9% compared to 39% in controls (OR, 1.74; 95% CI, 1.00–3.03; *p* = 0.051). Long-term reductions in UPCR were significant, with the voclosporin group maintaining a mean UPCR of 0.78 mg/mg versus 1.47 mg/mg in controls. Voclosporin also slowed the decline in eGFR, with a slope of −0.2 mL/min/1.73 m^2^/year versus −5.4 mL/min/1.73 m^2^/year in controls.

In light of the very quick reduction in proteinuria observed with voclosporin, an ideal target might be represented by heavily nephrotic patients, in which a prompt and persistent resolution of nephrotic-range proteinuria is desirable in order to prevent albuminuria-induced kidney function deterioration and/or the clinical complication of nephrotic syndrome (edema, thrombosis).

### 4.3. Obinutuzumab

In the phase 2 NOBILITY trial [13], 125 patients with proliferative LN were randomized to receive either obinutuzumab (1 g at week 0, 2, 24 and 26) or placebo in combination with SOC (MMF 2–2.5 g daily or equivalent doses of MPAA, 1–3 g methylprednisolone pulses followed by oral prednisone starting at 0.5 mg/kg daily tapered to 7.5 mg daily by week 12).

The primary endpoint of the study was the achievement of a complete renal response (CRR) at week 52, which was defined as a urine protein-to-creatinine ratio (UPCR) of less than 0.5 g/g, normal or near-normal renal function as indicated by a serum creatinine level no more than 15% higher than baseline and within the upper limit of normal, and inactive urinary sediment, characterized by fewer than 10 red blood cells per high-power field and the absence of red blood cell casts. Secondary endpoints included the proportion of patients achieving partial renal response (PRR), overall renal response (ORR, which includes both CRR and PRR), and modified CRR (mCRR), which required only UPCR under 0.5 g/g and normal serum creatinine levels without the sediment criteria.

At week 52, 35% of patients in the obinutuzumab group achieved CRR compared to 23% in the placebo group, although this difference did not reach statistical significance at that time (percentage difference: 12%, 95% CI: −3.4% to 28%, (*p* = 0.115)). However, by week 104, the CRR rate was significantly higher in the obinutuzumab group, with 41% achieving CRR compared to 23% in the placebo group (percentage difference: 19%, 95% CI: 2.7% to 35%, (*p* = 0.026)). Modified CRR at week 104 was also notably better in the obinutuzumab group, with 56% achieving mCRR compared to 34% in the placebo group (percentage difference: 22%, 95% CI: 5% to 39%, (*p* = 0.015)).

The NOBILITY trial further showed that the overall renal response (ORR) at weeks 52, 76, and 104 was consistently higher in the obinutuzumab group. At week 104, 54% of obinutuzumab-treated patients had achieved ORR, compared to only 29% in the placebo group (percentage difference: 25%, 95% CI: 8.2% to 42%, (*p* = 0.005)). In terms of renal function, patients receiving obinutuzumab had a significantly slower decline in eGFR, with an annualized eGFR slope advantage of 4.1 mL/min/1.73 m^2^ per year compared to placebo (95% CI: 0.14–8.08, (*p* = 0.043)).

Obinutuzumab reduced the risk of LN flare by 57% compared to placebo, with a hazard ratio (HR) of 0.43 (95% CI: 0.20–0.95). Additionally, obinutuzumab-treated patients experienced fewer sustained declines in kidney function, measured as a 30% or 40% reduction in eGFR from baseline. Specifically, the risk of a 30% eGFR decline was reduced by 80% (HR: 0.20, 95% CI: 0.06–0.61), and the risk of a 40% decline was reduced by 91% (HR: 0.09, 95% CI: 0.01–0.73).

A post hoc analysis [14] further supported the efficacy of obinutuzumab in maintaining kidney function and minimizing steroid use. By week 76, 38% of patients in the obinutuzumab group achieved CRR while maintaining a daily prednisone dose of 7.5 mg or less, compared to only 16% of patients in the placebo group (*p* < 0.01). Although the difference in steroid-sparing CRR was not statistically significant by week 104 (38% in the obinutuzumab group vs. 22% in the placebo group, (*p* = 0.06)), the cumulative steroid-sparing effect remained clinically meaningful.

In summary, the NOBILITY trial and its post hoc analysis demonstrated that obinutuzumab, when added to standard LN therapy, significantly improved complete and overall renal response rates, preserved kidney function, and reduced the risk of relapse.

Ongoing research aims to confirm these results. The Phase III REGENCY trial (NCT-04221477) has reported achieving its primary endpoint of complete renal response (CRR) at 76 weeks when comparing obinutuzumab to standard care for lupus nephritis (LN), as per a recent announcement (https://www.gene.com/media/press-releases/15038/2024-09-25/positive-phase-iii-results-for-genentech (accessed on: 13 January 2025)). Additionally, the ALLEGORY trial (NCT-04963296) is exploring the use of obinutuzumab in systemic lupus erythematosus (SLE), while the OBILUP study (NCT-04702256) is assessing the non-inferiority of obinutuzumab combined with MMF versus steroids and MMF, with findings expected by 2028.

### 4.4. Anifrolumab

Anifrolumab, a monoclonal antibody targeting the type I interferon (IFN) receptor subunit 1, is approved and recommended for SLE without LN, following the results of a phase 3 trial [23]. However, there is a rationale for its use in LN, stemming from the role of IFN pathways in LN pathogenesis, with over 80% of LN patients displaying elevated IFN gene signatures linked to active kidney disease and treatment resistance. In a phase II trial (TULIP-LN), patients with biopsy-proven active Class III or IV (+/− V) LN were randomized to receive either an intensified (IR, 900 mg initially followed by 300 mg) or basic regimen (BR, 300 mg) of anifrolumab, or placebo, alongside standard therapy with mycophenolate mofetil and glucocorticoids. At 52 weeks, the IR regimen demonstrated a numerically higher complete renal response (CRR) rate (45.5%) compared to placebo (31.1%), though the primary endpoint of relative proteinuria reduction was not met. Long-term data from the second year of the trial highlighted the safety and tolerability of anifrolumab, with the IR regimen maintaining superior renal and non-renal clinical outcomes, including proteinuria reduction, glucocorticoid tapering, and improved serological markers. These findings suggest that higher initial dosing (IR) is critical for achieving clinical efficacy in LN. Based on these results, further evaluation of anifrolumab in a phase III trial is ongoing [24,25]. Thus, its role in the management of LN is yet to be determined.

## 5. Racial Differences in Outcome and Refractory Lupus Nephritis

Understanding LN treatment outcomes across racial and ethnic groups is complex due to potential misclassification and the social dimensions of race. Data from the ALMS trial indicated that MMF and IVCY had similar efficacy in Asian and White patients. However, in Black and Hispanic patients, MMF demonstrated higher response rates compared to IVCY, with odds ratios of 2.4 (*p* = 0.03) and 2.5 (*p* = 0.011), respectively [26].

The AURORA study, involving a diverse cohort (32% Hispanic, 15% Black), showed consistent efficacy of voclosporin across subgroups [9]. In BLISS-LN, which included 14% Black patients, belimumab improved key renal outcomes in this group compared to placebo, despite overall lower response rates. On the other hand, Hispanic patients were under-represented in BLISS-LN [8].

Refractory LN typically refers to inadequate proteinuria or eGFR improvement after standard induction therapy. Treatment goals include progressively reducing proteinuria at 3, 6, and 12–24 months while maintaining eGFR stability. Persistent proteinuria might reflect chronic structural damage rather than an active disease, warranting a repeat kidney biopsy to guide treatment adjustments or identify coexisting conditions like thrombotic microangiopathy.

Management strategies include switching to CYC-based regimens, adding belimumab or CNIs to MMF, or considering rituximab, which has shown response rates exceeding 70% in observational studies [12]. Emerging options like obinutuzumab, complement inhibitors (e.g., eculizumab), and experimental therapies such as CAR T-cell or stem cell transplantation—which are beyond the scope of this review—are potential alternatives for severe cases [27]. Regular drug level monitoring can address nonadherence, a common issue in LN management, and improve treatment outcomes.

## 6. Controversies

The emergence of new therapies for LN raises two fundamental questions. First, since these drugs have been tested exclusively as add-on treatments to SOC, demonstrating efficacy benefits, they are shifting the traditional approach of a single immunosuppressant combined with glucocorticoids toward a model that incorporates multiple immunosuppressants while concurrently reducing overall steroid exposure. This paradigm shift is exemplified in findings from the AURORA trial, where voclosporin demonstrated substantial efficacy as part of a multitarget strategy that prioritized early reduction in corticosteroid doses.

Second, these treatments challenge the conventional two-phase approach of remission-induction followed by maintenance therapy. Instead, the tested regimens, including voclosporin and belimumab, are administered continuously across both phases, blurring the traditional distinction between induction and maintenance strategies [28].

A third critical aspect, which complicates the adoption of new therapies and applies equally to trials of well-established drugs like MMF, is the limited follow-up duration in clinical studies, typically around two years. Such timeframes are insufficient to capture hard renal outcomes, such as progression to ESKD. While reductions in proteinuria and stabilization of renal function are recognized surrogate markers, they do not necessarily correlate with long-term renal outcomes. Moreover, patients with reduced eGFR (<30 mL/min) were excluded from these trials, therefore limiting the generalizability of results to high-risk LN patients.

Specifically, although the BLISS-LN and the NOBILITY trials included patients with a new diagnosis of LN and no prior cyclophosphamide or MMF failure, i.e., patients at the onset of LN, they excluded patients with eGFR < 30 mL/min. The AURORA trial excluded patients with an eGFR less than 45 mL/min and allowed patients with a LN histological diagnosis up to two years before randomization, therefore potentially including patients that had already received partial disease control due to prior treatment. The multitarget trials on the other hand included also patients with low eGFR, but the population was exclusively Chinese, therefore again limiting the generalizability of results.

Therefore, there is a need for trials encompassing more diverse populations in terms of ethnicity and eGFR, capturing LN at its onset to avoid the confounding variable of prior treatment and an established long-standing diagnosis of SLE, which is per se an unfavorable prognostic marker, particularly with direct comparison between the new drugs on top of SOC.

Very few early markers of long-term renal outcomes are available in LN, namely a more than 50% proteinuria reduction at 6 months and a more than 25% proteinuria reduction at 12 weeks, which offered the rationale for proteinuria as a key outcome in these trials [29,30,31]. However, these cut-offs fail to capture patients who lose most of their GFR in the first weeks or months after LN onset and those who despite reductions in proteinuria eventually develop EKSD. Other markers, such as persistent C3 hypocomplementemia early in the course of the disease [28,32], might be good adjunctive surrogate markers of ESKD, but definitive answers regarding the effectiveness of new therapies for most at risk LN patients and their place in the management of the overall LN population will likely be available only with long follow up data.

## Figures and Tables

**Table 1:** Latest EULAR and KDIGO recommendations for the management of lupus nephritis.

Recommendations	EULAR 2023	KDIGO 2024
**All patients**	Hydroxychloroquine 5 mg/kg daily.	Hydroxychloroquine 5 mg/kg daily.
**Class I and II**	No specific recommendations.	Low-level proteinuria: guided by extrarenal manifestations. Nephrotic-range proteinuria: treated as minimal change disease (GC + CNIs).
**Proliferative Classes (III and IV) with or without a membranbous (class V) component**	Glucocorticoids with MMF or low-dose IV CyC. High-risk patients: IV CyC high dose (NIH). Maintenance: MPAA preferred.	Glucocorticoids with MPAA, low-dose IV CyC, belimumab + low-dose IV CyC, or MPAA + CNI. Maintenance: MPAA preferred.
**High-Risk Patients**	Crescents, fibrinoid necrosis, severe interstitial inflammation, or eGFR < 45 mL/min/1.73 m^2^.	Similar considerations implied for refractory or severe cases.
**Class V**	Tailored to the individual patient characteristic, and no immunosuppression unless proteinuria > 1 g/24 h despite RAAS blockade.	GC with MPAA or low-dose IV CyC recommended if nephrotic range proteinuria despite RAAS blockade; same regimen can be considered when proteinuria worsens and/or its complications arise.
**Glucocorticoids**	IV MP pulses (250–1000 mg/day, 1–3 days), followed by PO (0.3–0.5 mg/kg/day, tapered). Maintenance: ≤5 mg/day prednisone.	IV MP pulses (0.25–0.5 g/day, 1–3 days), followed by PO (0.35–1.0 mg/kg/day, max 80 mg) tapered over 11–21 weeks.
**Rituximab**	Should be considered for organ- or life-threatening manifestations.	Should be considered for standard-of-care refractory cases.
**Cyclophosphamide**	Low dose (EuroLupus): 500 mg at weeks 0, 2, 4, 6, 8 (total 2.5 g). High dose (NIH): 0.75–1.0 g/m^2^ at weeks 0, 4, 8, 12, 16, 20 (9–12 g).	Same as EULAR.
**MPAA (Mycophenolic Acid Analogs)**	Induction: MMF 2–3 g/day; MPA 1440–2160 mg/day. Maintenance: MMF 1–2 g/day; MPA 720–1440 mg/day.	Same as EULAR.
**Drug Discontinuation**	Treatment duration: 2–3 years. GCs withdrawn first, then immunosuppressives after 2 years of stable remission.	Treatment duration: ≥3 years. GCs withdrawn only after ≥1 year of stable remission.

## References

[1] Siegel C.H., Sammaritano L.R. (2024). Systemic Lupus Erythematosus: A Review. JAMA.

[2] Austin H.A., Klippel J.H., Balow J.E., le Riche N.G., Steinberg A.D., Plotz P.H., Decker J.L. (1986). Therapy of lupus nephritis. Controlled trial of prednisone and cytotoxic drugs. N. Engl. J. Med..

[3] Houssiau F.A., Vasconcelos C., D’Cruz D., Sebastiani G.D., Garrido E.d.R., Danieli M.G., Abramovicz D., Blockmans D., Mathieu A., Direskeneli H. (2002). Immunosuppressive therapy in lupus nephritis: The Euro-Lupus Nephritis Trial, a randomized trial of low-dose versus high-dose intravenous cyclophosphamide. Arthritis Rheum..

[4] Ginzler E.M., Dooley M.A., Aranow C., Kim M.Y., Buyon J., Merrill J.T., Petri M., Gilkeson G.S., Wallace D.J., Weisman M.H. (2005). Mycophenolate mofetil or intravenous cyclophosphamide for lupus nephritis. N. Engl. J. Med..

[5] Dooley M.A., Jayne D., Ginzler E.M., Isenberg D., Olsen N.J., Wofsy D., Eitner F., Appel G.B., Contreras G., Lisk L. (2011). Mycophenolate versus azathioprine as maintenance therapy for lupus nephritis. N. Engl. J. Med..

[6] Liu Z., Zhang H., Liu Z., Xing C., Fu P., Ni Z., Chen J., Lin H., Liu F., He Y. (2015). Multitarget therapy for induction treatment of lupus nephritis: A randomized trial. Ann. Intern. Med..

[7] Zhang H., Liu Z., Zhou M., Liu Z., Chen J., Xing C., Lin H., Ni Z., Fu P., Liu F. (2017). Multitarget Therapy for Maintenance Treatment of Lupus Nephritis. J. Am. Soc. Nephrol..

[8] Furie R., Rovin B.H., Houssiau F., Malvar A., Teng Y.K.O., Contreras G., Amoura Z., Yu X., Mok C.-C., Santiago M.B. (2020). Two-Year, Randomized, Controlled Trial of Belimumab in Lupus Nephritis. N. Engl. J. Med..

[9] Rovin B.H., Teng Y.K.O., Ginzler E.M., Arriens C., Caster D.J., Romero-Diaz J., Gibson K., Kaplan J., Lisk L., Navarra S. (2021). Efficacy and safety of voclosporin versus placebo for lupus nephritis (AURORA 1): A double-blind, randomised, multicentre, placebo-controlled, phase 3 trial. Lancet.

[10] Saxena A., Ginzler E.M., Gibson K., Satirapoj B., Santillán A.E.Z., Levchenko O., Navarra S., Atsumi T., Yasuda S., Chavez-Perez N.N. (2024). Safety and Efficacy of Long-Term Voclosporin Treatment for Lupus Nephritis in the Phase 3 AURORA 2 Clinical Trial. Arthritis Rheumatol..

[11] Rovin B.H., Furie R., Latinis K., Looney R.J., Fervenza F.C., Sanchez-Guerrero J., Maciuca R., Zhang D., Garg J.P., Brunetta P. (2012). Efficacy and safety of rituximab in patients with active proliferative lupus nephritis: The Lupus Nephritis Assessment with Rituximab study. Arthritis Rheum..

[12] Weidenbusch M., Römmele C., Schröttle A., Anders H.-J. (2013). Beyond the LUNAR trial. Efficacy of rituximab in refractory lupus nephritis. Nephrol. Dial. Transplant..

[13] Furie R.A., Aroca G., Cascino M.D., Garg J.P., Rovin B.H., Alvarez A., Fragoso-Loyo H., Zuta-Santillan E., Schindler T., Brunetta P. (2022). B-cell depletion with obinutuzumab for the treatment of proliferative lupus nephritis: A randomised, double-blind, placebo-controlled trial. Ann. Rheum. Dis..

[14] Rovin B.H., Furie R.A., Ross Terres J.A., Giang S., Schindler T., Turchetta A., Garg J.P., Pendergraft W.F., Malvar A. (2024). Kidney Outcomes and Preservation of Kidney Function with Obinutuzumab in Patients with Lupus Nephritis: A Post Hoc Analysis of the NOBILITY Trial. Arthritis Rheumatol..

[15] Eudy A.M., Clowse M.E., Corneli A., McKenna K., Pisetsky D.S., Maheswaranathan M., Burshell D., Doss J., Sun K., Sadun R.E. (2024). The Type 1 & 2 systemic lupus erythematosus model: Perspectives of people living with systemic lupus erythematosus. Lupus.

[16] Fanouriakis A., Tziolos N., Bertsias G., Boumpas D.T. (2021). Update οn the diagnosis and management of systemic lupus erythematosus. Ann. Rheum. Dis..

[17] Hoi A., Igel T., Mok C.C., Arnaud L. (2024). Systemic lupus erythematosus. Lancet.

[18] Fanouriakis A., Kostopoulou M., Andersen J., Aringer M., Arnaud L., Bae S.-C., Boletis J., Bruce I.N., Cervera R., Doria A. (2024). EULAR recommendations for the management of systemic lupus erythematosus: 2023 update. Ann. Rheum. Dis..

[19] Rovin B.H., Ayoub I.M., Chan T.M., Liu Z.H., Mejía-Vilet J.M., Floege J. (2024). Kidney Disease: Improving Global Outcomes (KDIGO) Lupus Nephritis Work Group KDIGO 2024 Clinical Practice Guideline for the management of LUPUS NEPHRITIS. Kidney Int..

[20] Levy R.A., Gonzalez-Rivera T., Khamashta M., Fox N.L., Jones-Leone A., Rubin B., Burriss S.W., Gairy K., van Maurik A., Roth D.A. (2021). 10 Years of belimumab experience: What have we learnt?. Lupus.

[21] Rovin B.H., Furie R., Teng Y.K.O., Contreras G., Malvar A., Yu X., Ji B., Green Y., Gonzalez-Rivera T., Bass D. (2022). A secondary analysis of the Belimumab International Study in Lupus Nephritis trial examined effects of belimumab on kidney outcomes and preservation of kidney function in patients with lupus nephritis. Kidney Int..

[22] van Gelder T., Lerma E., Engelke K., Huizinga R.B. (2022). Voclosporin: A novel calcineurin inhibitor for the treatment of lupus nephritis. Expert. Rev. Clin. Pharmacol..

[23] Morand E.F., Furie R., Tanaka Y., Bruce I.N., Askanase A.D., Richez C., Bae S.-C., Brohawn P.Z., Pineda L., Berglind A. (2020). Trial of Anifrolumab in Active Systemic Lupus Erythematosus. N. Engl. J. Med..

[24] Jayne D., Rovin B., Mysler E.F., Furie R.A., Houssiau F.A., Trasieva T., Knagenhjelm J., Schwetje E., Chia Y.L., Tummala R. (2022). Phase II randomised trial of type I interferon inhibitor anifrolumab in patients with active lupus nephritis. Ann. Rheum. Dis..

[25] Jayne D., Rovin B., Mysler E., Furie R., Houssiau F., Trasieva T., Knagenhjelm J., Schwetje E., Tang W., Tummala R. (2023). Anifrolumab in lupus nephritis: Results from second-year extension of a randomised phase II trial. Lupus Sci. Med..

[26] Influence of Race/Ethnicity on Response to Lupus Nephritis Treatment: The ALMS Study—PubMed. https://pubmed.ncbi.nlm.nih.gov/19933596/.

[27] Parodis I., Depascale R., Doria A., Anders H.-J. (2024). When should targeted therapies be used in the treatment of lupus nephritis: Early in the disease course or in refractory patients?. Autoimmun. Rev..

[28] De Vriese A.S., Sethi S., Fervenza F.C. (2024). Lupus nephritis: Redefining the treatment goals. Kidney Int..

[29] Dall’Era M., Levesque V., Solomons N., Truman M., Wofsy D. (2015). Identification of clinical and serological factors during induction treatment of lupus nephritis that are associated with renal outcome. Lupus Sci. Med..

[30] Tamirou F., Lauwerys B.R., Dall’Era M., Mackay M., Rovin B., Cervera R., Houssiau F.A., MAINTAIN Nephritis Trial Investigators (2015). A proteinuria cut-off level of 0.7 g/day after 12 months of treatment best predicts long-term renal outcome in lupus nephritis: Data from the MAINTAIN Nephritis Trial. Lupus Sci. Med..

[31] Dall’Era M., Cisternas M.G., Smilek D.E., Straub L., Houssiau F.A., Cervera R., Rovin B.H., Mackay M. (2015). Predictors of long-term renal outcome in lupus nephritis trials: Lessons learned from the Euro-Lupus Nephritis cohort. Arthritis Rheumatol..

[32] Rossi G.M., Maggiore U., Peyronel F., Fenaroli P., Delsante M., Benigno G.D., Gianfreda D., Urban M.L., Manna Z., Arend L.J. (2022). Persistent Isolated C3 Hypocomplementemia as a Strong Predictor of End-Stage Kidney Disease in Lupus Nephritis. Kidney Int. Rep..

